# Semantics-Based Composition of Integrated Cardiomyocyte Models Motivated by Real-World Use Cases

**DOI:** 10.1371/journal.pone.0145621

**Published:** 2015-12-30

**Authors:** Maxwell L. Neal, Brian E. Carlson, Christopher T. Thompson, Ryan C. James, Karam G. Kim, Kenneth Tran, Edmund J. Crampin, Daniel L. Cook, John H. Gennari

**Affiliations:** 1 Department of Biomedical Informatics and Medical Education, University of Washington, Seattle, Washington, United States of America; 2 Department of Molecular and Integrative Physiology, University of Michigan, Ann Arbor, MI, United States of America; 3 Auckland Bioengineering Institute, The University of Auckland, Auckland, New Zealand; 4 Systems Biology Laboratory, Melbourne School of Engineering, University of Melbourne, Victoria, Australia; 5 ARC Centre of Excellence in Convergent Bio-Nano Science and Technology, Melbourne School of Engineering, University of Melbourne, Victoria, Australia; 6 School of Mathematics and Statistics, University of Melbourne, Victoria, Australia; 7 School of Medicine, University of Melbourne, Victoria, Australia; 8 Department of Physiology and Biophysics, University of Washington, Seattle, WA, United States of America; University of Illinois-Chicago, UNITED STATES

## Abstract

Semantics-based model composition is an approach for generating complex biosimulation models from existing components that relies on capturing the biological meaning of model elements in a machine-readable fashion. This approach allows the user to work at the biological rather than computational level of abstraction and helps minimize the amount of manual effort required for model composition. To support this compositional approach, we have developed the SemGen software, and here report on SemGen’s semantics-based merging capabilities using real-world modeling use cases. We successfully reproduced a large, manually-encoded, multi-model merge: the “Pandit-Hinch-Niederer” (PHN) cardiomyocyte excitation-contraction model, previously developed using CellML. We describe our approach for annotating the three component models used in the PHN composition and for merging them at the biological level of abstraction within SemGen. We demonstrate that we were able to reproduce the original PHN model results in a semi-automated, semantics-based fashion and also rapidly generate a second, novel cardiomyocyte model composed using an alternative, independently-developed tension generation component. We discuss the time-saving features of our compositional approach in the context of these merging exercises, the limitations we encountered, and potential solutions for enhancing the approach.

## Introduction

While biomedical researchers now have the computational power to express complex hypotheses about biological systems using quantitative biosimulation models, repurposing previously-published models to compose larger, multiscale systems remains a largely manual, time-consuming and error-prone task. Few tools exist that help automate the process of merging models into more complex, biologically consistent systems. Developing such tools is challenging because, for example, modelers do not all encode their models in the same language and they do not always employ the same system of physical units. Another critical challenge is that systems-level hypotheses differ between research groups; two modelers may conceptualize the same system at different levels of granularity, with different sets of biological role players, and with different input/output arrangements between interacting components. Harmonizing these differences at the time of model merging is often cumbersome. Thus, there is a need for a flexible model composition approach that accommodates the various ways researchers conceptualize a biological system, and one that scales across modeling languages [1]. To meet these needs we have developed the SemSim (semantic simulation) model description format [2,3], which describes a biosimulation model in terms of its biological meaning formally linked to the model’s specific mathematical implementation. To accomplish this we use composite annotations [2], a set of structured statements that link controlled ontology terms to form a precise, logical definition of what is being simulated. We currently encode SemSim models in OWL [4], but other knowledge representation formats might be used.

Extraction and merging of biosimulation models begins by annotating each model using our Java-based application, SemGen, available at http://sbp.bhi.washington.edu/projects/semgen. SemGen provides utilities for annotating existing models encoded in CellML [5], Systems Biology Markup Language (SBML) [6] and JSim’s Mathematical Modeling Language (MML) [7] as well as utilities for extracting out parts of models and merging models into new systems. Our goal with SemGen is to provide a tool that facilitates biological annotation of a wide variety of available models and allows modelers to perform composition tasks at the biological, rather than computational, level of abstraction. That is, modelers who wish to merge two models will not necessarily need to have code-level knowledge of the models to couple them into a biologically-consistent system.

One of our primary objectives is to provide modelers with a suite of tools that will allow them to more easily share, repurpose, and compose multiscale models of physiological systems. We have previously demonstrated SemGen’s merging capabilities using example model composition tasks derived from our own work as multiscale physiological modelers [2,3,8]. Our aim for the present study was to test these capabilities further using more complex merging tasks derived from external, real-world modeling efforts in physiology. We identified the “Pandit-Hinch-Niederer” (PHN) model [9,10], an integrated cardiomyocyte composition, as a candidate merging task, given its level of complexity and relevance to multiscale cardiac physiology. For this study we aimed to reproduce the PHN composition using SemGen and also further stress-test the application by creating variations of the PHN model using alternative source models.

**Fig 1:**
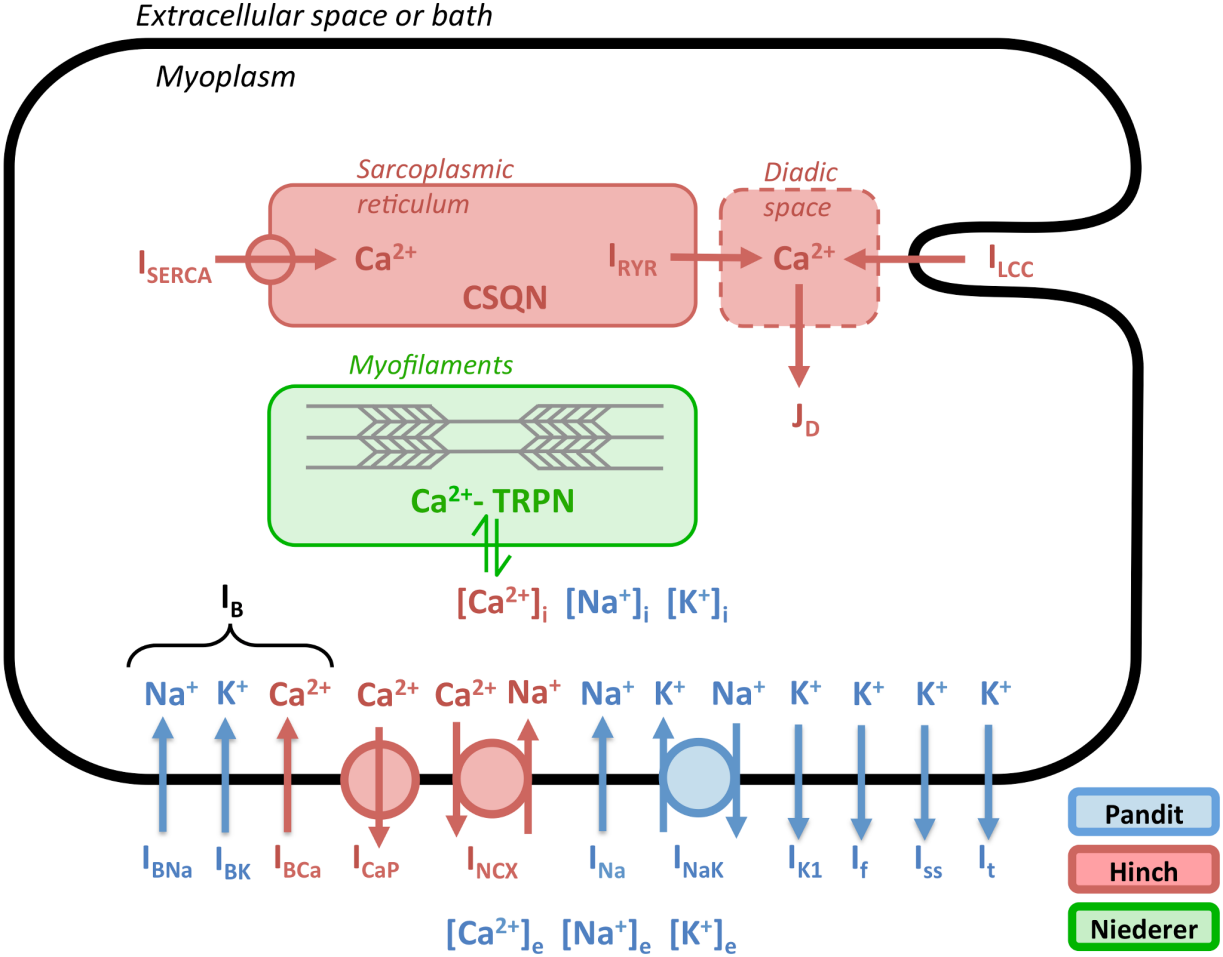
Architecture of the integrated PHN model. Coloring indicates a component’s source model. Ion flows without circles represent facilitated transport; those with circles represent active transport. Adapted from Fig 1 of Terkildsen et al. [9].

The original design and creation of the PHN model is described in Terkildsen et al. [9]. To summarize, Niederer and Smith [10] first reported the integration of this model, and then Terkildsen et al. re-created the integration task to showcase how a composite model can be created from independent CellML components that are imported by reference into a parent CellML 1.1 model. The Terkildsen et al. version was created by first merging the electrophysiology model of Pandit et al. [11] (referred to here as the “Pandit” model) with the calcium dynamics model of Hinch et al. [12] (referred to here as the “Hinch” model). Then, the active tension development model of Niederer et al. [13] (referred to here as the “Niederer” model) was incorporated to connect intracellular calcium dynamics to tension generated by the cell. The result is a cardiomyocyte model that integrates electrophysiological dynamics, calcium-induced calcium release, and active tension development (Fig 1). The CellML version of the PHN model published by Terkildsen et al. is available at https://models.cellml.org/exposure/e8ee336095b8955f75a3e6c09b791d42/Pandit_Hinch_Niederer.cellml/view.

Using the Terkildsen et al. publication as a guide, we attempted to reproduce the PHN model by first downloading the source models from the CellML repository, annotating their biological content according to the SemSim framework, merging the Pandit and Hinch models with SemGen’s Merger tool [2], then merging the result with the Niederer model. Ultimately, we were able to generate a merged system that faithfully reproduces the numerical results of the PHN model published by Terkildsen et al. including membrane voltage, calcium dynamics and active tension development. Here we report the results from our PHN merging exercise, as well as some important limitations we identified that, if resolved, would have allowed further automation of the modeling task and obviated some code-level management required for model-to-model coupling. Most importantly, we discuss the need for a semantic-similarity scoring metric that will allow SemGen to recognize semantically similar, not just identical, biological content shared between models.

We also report here on a follow-up test of our semantics-based modeling approach where we coupled the Pandit-Hinch merged system to a different model of active tension development based on Tran et al. [14] This tension model (referred to here as the “Tran” model) is distinct from the Niederer model in that it includes the effects of ATP, ADP, phosphate and pH on force development. This model-merging task was first performed at a conference as an impromptu collaboration between three of the authors. We describe this initial collaboration here as supplementary evidence, albeit anecdotal, that demonstrates the time-saving features of our semantics-based modeling approach.

## Materials and Methods

In this section we describe the methodology behind SemGen’s semantics-based merging tool as well as the approach we used to annotate the source models used in our integrated cardiomyocyte models, including several “semantic alignment” steps required for SemGen to automatically recognize key points of semantic overlap between the models. We then describe the specific steps performed to couple the models within SemGen and generate simulation output.

### SemGen’s Merger Tool

We designed SemGen to support a particular style of model merging, where the interface between the source models to be merged is formed at the time of merging and is based on the biophysical overlap between the models. We refer to this approach as “semantics-based, adaptable interface modularity” (SAIM) [1]. We contend that this approach is more scalable in the context of community-wide model sharing than pre-defining model-to-model interfaces because such interfaces may only be useful to research groups that share a common conceptualization of how a biological system should be organized for study. Because such conceptualizations differ between research groups, it is instead more practical to form the links between shared models based on their biological content, rather than specific input/output designations within the code.

When two models are loaded into the Merger tool, SemGen analyzes their semantic annotations and lists the biophysical concepts shared by both models. Then, the user decides which mathematical implementation of the concept should be preserved in the merged system (or to ignore the equivalency and preserve both representations in the merged system). Once this resolution process is completed, SemGen makes the appropriate computational links between the models to couple them. Thus, it is crucial that models are annotated in a consistent fashion such that SemGen can recognize biological equivalencies between the models. To promote this consistency, we have developed an annotation protocol described next.

### Model Annotation

We annotated the source models of our integrated cardiomyocyte simulations as part of a broader effort to create SemSim versions of a substantial set of publicly available CellML models. In order to test the ability of SemGen to combine model components annotated by different individuals, multiple annotators were assigned non-overlapping sets of CellML models to annotate in SemGen, including the Pandit, Hinch, and Niederer models. One author annotated the Pandit model and another annotated the Hinch and Niederer models. Both of these authors collaborated on annotating the Tran model. To promote inter-annotator consistency, we developed an annotation protocol before embarking on our annotation effort. Here we briefly describe our protocol for capturing the biological meaning of a model’s contents within SemSim models.

The existing set of biophysical concepts in the corpus of publicly available ontologies is not detailed enough to provide single controlled terms as annotations for the physical properties often represented in biosimulation models. For example, a concept common to all three of the Pandit, Hinch and Niederer models is cytosolic calcium concentration in the cardiomyocyte; however, to our knowledge no single ontology term exists that represents this concept. To create a logical, machine-readable definition for this concept, we decompose the concept into a sequence of multiple ontology terms, linked via standardized ontological relationships. We call this post-coordinated construct a “composite annotation” [2]. The composite annotation for this particular example links the physical property “concentration” to a partonomy of multiple physical entities: calcium, cytosol, and cardiomyocyte.

In our annotation approach we distinguish among three types of physical properties represented in biosimulation models: properties of physical entities (concentrations, volumes, etc.), properties of physical processes (chemical reaction flow rates, electrical currents, etc.), and properties of constitutive dependencies (chemical reaction rate constants, vascular compliances, etc.). We make these distinctions based on the logical, formal organization of the physical property hierarchy in the Ontology of Physics for Biology (OPB—[15,16]). Rather than annotate every physical concept in a biosimulation model, we instead focus on the first two types: properties of entities and processes. We have found that when performing semantics-based merging, the coupling points between models predominantly involve these properties, and coupling the models at these points subsequently resolves most, if not all, of the semantic overlap between the constitutive properties shared by the models.

#### Annotating properties of physical entities

To annotate physical entities, we created composite annotations as described in our cytosolic calcium concentration example above. In each case we used terms from the OPB for the physical property component of the composite annotation. This was done because the OPB provides adequate coverage and principled organization of physical property concepts commonly used in biosimulation models. This principled organization is critical for reaching several of our broader research goals: performing automated reasoning over SemSim models to support advanced search and retrieval of models [17], further automating model annotation, detecting semantic similarities between models, and identifying biophysical inconsistencies in models. The OPB is being developed in close collaboration with SemGen so that we can leverage the ontology’s content and organization to support these broader research goals.

For the physical entity components of our composite annotations, we have relied on a limited subset of available ontologies and terminologies as resources for naming and referencing physical entities in composite annotations. We selected this subset of ontologies, shown in Table 1, to promote inter-annotator consistency when creating composite annotations. We rely primarily on the Foundational Model of Anatomy (FMA—[18]) for anatomical terms because of its deep coverage, principled organization, and mereotopological detail. Although the FMA is comprehensive in its coverage of macroscopic human anatomy, we use the Mouse Adult Gross Anatomy Ontology (MA—[19]) for rodent-specific anatomical structures (e.g., tail vein), the Cell Type Ontology (CL—[20]) for non-mammalian cell types, and the cellular component branch of the Gene Ontology (GO:cc—[21]) for macromolecular structures not represented in the FMA. At the molecular level, we use the Protein Ontology (PR—[22]) for proteins and the Chemical Entities of Biological Interest (ChEBI—[23]) resource for atoms and small molecules. For laboratory materials such as cell culture media and patch clamps, we use the Ontology for Biomedical Investigations (OBI—[24]).

**Table 1:** The set of knowledge resources from which we selected terms to use in our composite annotations for properties of physical entities.

Knowledge resource	Scope of use
Foundational Model of Anatomy (FMA)	Macromolecular to organism-level anatomy
Mouse Adult Gross Anatomy (MA)	Rodent-specific anatomy
Cell Ontology (CL)	Non-mammalian cell types
Gene Ontology:cellular component (GO:cc)	Macromolecular structures not represented in FMA
Protein Ontology (PR)	Proteins
Chemical Entities of Biological Interest (ChEBI)	Atoms and small molecules
Ontology for Biomedical Investigations (OBI)	Laboratory materials

When annotating our source models, we sometimes found that a precise reference term for a physical entity was not available. In such cases we created custom physical entities for use in the model. For example, the Niederer model simulates the cytosolic concentration of calcium-bound troponin. Because calcium-bound troponin is not a concept represented in any publicly available knowledge resource we know of, we introduced a custom term within the model. Although such custom terms are sometimes unavoidable, they do present a challenge for recognizing the semantic overlap between models. Because they are not defined against reference terms, SemGen currently has no way to determine the biological meaning of a custom physical entity, and therefore cannot recognize inter-model semantic equivalencies for composite annotations that use custom physical entity terms. We discuss this important limitation and its impact on our ability to automate the PHN merging operation later in the Discussion section.

#### Annotating properties of physical processes

When creating composite annotations for physical processes, we use a logical structure that is different than for physical entities. For each process property, we select the OPB property that is represented (electrical current, for example), and link it to a custom term in SemGen that represents the process itself. This is done because no multiscale ontology of physical processes currently exists that formally connects processes to the physical entities that participate in them. Such connections are critical for our model merging purposes because merging often requires reformulating conservation equations (e.g. mass balance laws) that define the amount of a physical entity in a system. Automating such reformulations would require that SemGen know which processes consume and produce which physical entities. No existing process ontology provides this information, and although the OPB has been designed to eventually represent a set of physical process types, specifying the entity participants in those processes is beyond its scope. Therefore, our approach is to create a custom physical process term within SemGen for each process property, and explicitly designate which entities are consumed, produced or otherwise influence the process. To promote consistency and reduce effort when annotating the properties of physical processes, we annotated them only after annotating the properties of physical entities. This way we were able to readily specify process participants by selecting them from the set of physical entities already added to the model.

To identify semantic equivalencies between properties of processes in models, SemGen analyzes the specific physical property represented, and the entities that participate in the process. If the property and the participating entities are all semantically equivalent by virtue of their ontological annotations, SemGen identifies the processes as equivalent.

#### Annotating constitutive properties

As mentioned above, we did not attempt to annotate all the constitutive properties in the Pandit, Hinch, Niederer and Tran models; such annotations would have provided little benefit to the model merging process. However, some constitutive properties are available as single reference terms in the OPB due to widespread use in biophysical modeling. The universal gas constant and Faraday’s constant are two such concepts present in the Pandit and Hinch models that we annotated using single reference terms (as opposed to composites) from the OPB. At the time of model merging, SemGen compares singular as well as composite annotations; therefore, the program can identify semantic equivalencies based on singular reference terms such as these and can notify the user of differences in their computational implementation, including physical unit assignments.

### Semantic Alignment of Ion Channel Activity

As reported in Terkildsen et al., there is a crucial difference between the Pandit and Hinch models that must be addressed before they can be coupled in a biophysically consistent way: ion channel activity rates are expressed as electrical currents (in nanoamps) in the Pandit model, and as chemical concentration flow rates (in millimolar/sec) in the Hinch model. Therefore, Terkildsen et al. added additional “glue code” to their merged system that converts the chemical concentration flow rates into currents. Because current is semantically distinct from chemical concentration flow rate, we had to perform a similar alignment between the models for SemGen to automatically recognize the ion channel commonalities shared by the Pandit and Hinch models. To address this issue we copied in the computational block of glue code that Terkildsen et al. added into the Hinch source model, and used this new version of the model in our annotation and merging processes.

### Merging Models in SemGen

After annotating our source models, we performed sequential merges, two models at a time, within SemGen’s Merger tool. After analyzing the semantic annotations, SemGen presented the set of matching annotations present in the two models along with the model codewords associated with them. (As mentioned above, these matching annotations indicate the biologically equivalent concepts among the two models). We then selected which mathematical formulation of those biological concepts we wished to preserve in the merged model based on the composition described by Terkildsen et al.

SemGen was able to automatically identify many, but not all, of the biological equivalencies during the merging process. Left unresolved, these unrecognized equivalencies result in redundant biological content being copied into the merged model. Therefore, for those cases where we knew SemGen had failed to find an equivalency, we determined whether we needed to manually specify the equivalency so that we could appropriately couple the models, computationally. In some cases the presence of redundant content may not impact the variables that a user cares about simulating; while the content is computed in the merged model, it may be a computational dead end having no downstream mathematical impact on any of the variables of interest. In other cases, copying in the redundant content results in two different mathematical formulations of the same biophysical property being used to compute a variable of interest; a logical inconsistency in the model that must be avoided. Therefore, for each equivalency not identified by SemGen, our challenge was to determine whether ignoring the equivalency (and thus copying in redundant mathematical content) would create such a logical inconsistency or would simply generate inconsequential content that could be manually pruned out later. If the former, then we manually specified the equivalency at the time of merging and resolved it according to the decisions made by Terkildsen et al. Creating the PHN model required two instances of this kind of manual intervention. See Tables 2 and 3 for the specific instances.

**Table 2:** Semantic equivalencies between the annotated Pandit and Hinch models grouped according to whether SemGen identified them or not.

Shared biophysical property	Resolution decision
*Automatically identified by SemGen*	
Background calcium current	Hinch
Calcium-ATPase pump current	Hinch
Sodium/calcium exchanger current	Hinch
Intracellular calcium ion concentration	Hinch
Intracellular sodium ion concentration	Pandit
Extracellular calcium concentration	Pandit
Extracellular sodium concentration	Pandit
Membrane voltage	Pandit
Cytosolic volume	Hinch
Ambient temperature	Hinch
Universal gas constant	Hinch
Faraday constant	Hinch
Temporal solution domain	Hinch
*Not identified*	
* L-type calcium channel current	Hinch
Ryanodine receptor current	Unresolved
SERCA pump current	Unresolved
Troponin-calcium buffering rate	Unresolved
Diadic space calcium concentration	Unresolved
Sarcoplasmic reticulum calcium concentration	Unresolved
Concentration of bound and unbound calmodulin	Unresolved
Concentration of bound and unbound troponin	Unresolved
Troponin-calcium association rate constant	Unresolved
Troponin-calcium dissociation rate constant	Unresolved
Calmodulin-calcium rapid buffer coefficient	Unresolved

*Required a manual mapping between models to produce desired PHN simulation results.

**Table 3:** Semantic equivalencies between the merged Pandit-Hinch model and the Niederer model grouped according to whether SemGen identified them or not.

Shared biophysical property	Resolution decision
*Automatically identified by SemGen*	
Intracellular calcium ion concentration	Pandit-Hinch
Intracellular troponin concentration (Hinch and Niederer representations)	Niederer
Temporal solution domain	Pandit-Hinch
*Not identified*	
* Troponin-calcium buffering rate (shared by Pandit, Hinch and Niederer)	Niederer
Concentration of bound and unbound troponin (shared by Pandit, Hinch and Niederer)	Unresolved
Troponin-calcium association rate constant (shared by Pandit, Hinch and Niederer)	Unresolved
Troponin-calcium dissociation rate constant (shared by Pandit, Hinch and Niederer)	Unresolved

*Required a manual mapping between models to reproduce PHN simulation results.

After resolving all biological equivalencies, SemGen prompts the user to provide new names to disambiguate any variables and submodels that have identical names in the two models to be merged. SemGen also recognizes unit inconsistencies between model variables that it identifies as semantically equivalent. For example, myoplasmic volume is expressed in microliters in the Pandit model, but in cubic micrometers in the Hinch model. However, we were able to ignore prompts warning of unit inconsistencies during the merging process because the two simulation platforms we planned to use for validating our merged model—JSim [7] and OpenCOR [25]—support automatic unit conversion. Once our final merged SemSim models were generated, we converted them into CellML using SemGen’s SemSim-to-CellML translation function and simulated the model in JSim and OpenCOR.

To gauge SemGen’s ability to merge models in a biologically consistent fashion, we also compared the points of semantic overlap identified by SemGen during merging against the points identified by a human expert. Based on this comparison, we discuss potential solutions for improving SemGen’s ability to identify biological overlap points between models. We outline these solutions in the Discussion section.

### Substituting the Tran Force-Generation Model for the Niederer Model

The Tran model of cardiomyocyte crossbridge force development is based on the model by Rice et al. [26] and adds the regulation of crossbridge kinetics as a function of metabolites such as ATP and ADP. The purpose of merging the Tran model to the existing Pandit-Hinch merge is to demonstrate that functional model components (in this case force production) can be swapped into or out of larger composite models. For that reason, the link we made between the two models was through the cytosolic calcium concentration only, and we did not replace the buffering of calcium by troponin in the Pandit-Hinch model with the calcium-troponin binding in the Tran model. Three of the authors (KT, BEC and JHG) performed the initial merging attempt with the Tran model as an impromptu exercise at a conference. They were able to generate a runnable simulation from SemGen in approximately 15 minutes. Following-up on this initial attempt, we re-performed the PHT merge using a more thoroughly annotated version of the Tran model, the results of which are reported here.

### Validation of Merged Models

To validate our version of the PHN model, we compared our simulation results for intracellular calcium, membrane voltage, and active myocyte tension to those from the model published by Terkildsen et al. We then investigated the mathematical differences between the versions that accounted for any deviations between the results. To validate the PHT model, we investigated whether the model gave expected results for cardiomyocyte force generation given the intracellular calcium and membrane dynamics provided by the Pandit-Hinch model.

## Results

### Pandit-Hinch-Niederer Merge

Table 2 shows which semantic equivalencies SemGen automatically identified when merging the Pandit and Hinch models based on the variables’ annotations. In cases where SemGen did not identify an existing equivalency where it should have, we determined whether we needed to manually specify the equivalency so that we could generate a merged PHN model that would correctly compute our variables of interest. These manually-specified equivalencies are indicated by an asterisk in Tables 2, 3 and 4.

**Table 4:** Semantic equivalencies between the merged Pandit-Hinch model and the Tran model grouped according to whether SemGen identified them or not.

Shared biophysical property	Resolution decision
*Automatically identified by SemGen*	
Intracellular calcium ion concentration	Pandit-Hinch
Ambient temperature	Pandit-Hinch
Temporal solution domain	Pandit-Hinch
*Not identified*	
Concentration of bound and unbound troponin (shared by Pandit, Hinch and Tran)	Unresolved
Concentration of calcium-bound troponin (shared by Pandit and Tran)	Unresolved
Troponin-calcium buffering rate (shared by Pandit, Hinch and Tran)	Unresolved
Troponin-calcium association rate constant (shared by Pandit, Hinch and Tran)	Unresolved
Troponin-calcium dissociation rate constant (shared by Pandit, Hinch and Tran)	Unresolved

Table 3 shows which semantic equivalencies SemGen automatically identified when merging the Pandit-Hinch and Niederer models as well as the equivalencies that we had to manually specify and those that we left unresolved.

**Fig 2:**
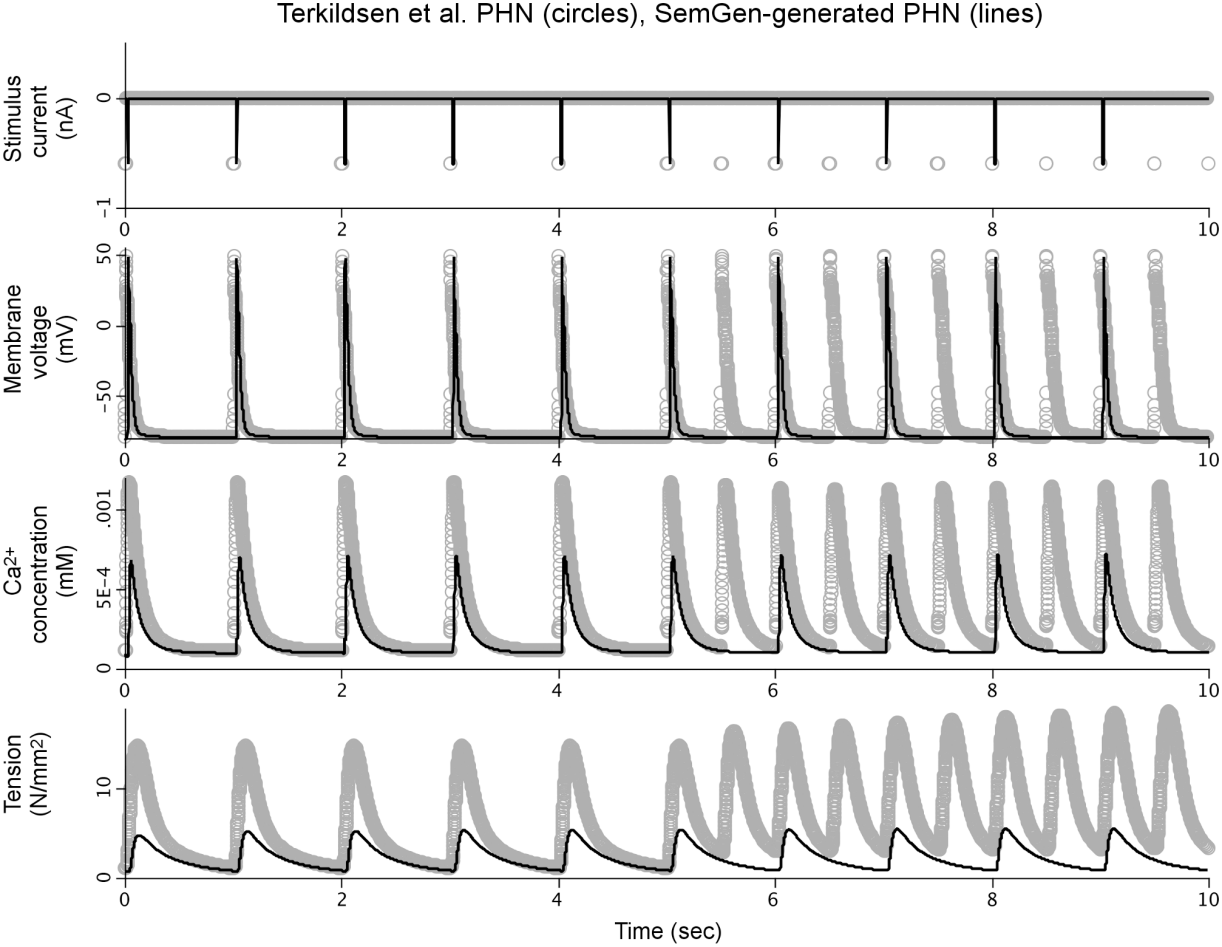
Comparison between simulation results from the original PHN model and the SemGen-generated version.

Fig 2 compares simulation results from our SemGen-generated PHN model against those of the Terkildsen et al. PHN model. The results show that we were able to couple the models such that the membrane stimulation current used in the Pandit model creates an intracellular calcium transient. These calcium dynamics are controlled by the Hinch calcium mass balance equations, and they in turn produce active myocyte tension development according to the Niederer model formulation.

We investigated a number of significant numerical differences between the two versions of the model and found that some of the computational aspects from the Pandit, Hinch and Niederer source models were adjusted in the Terkildsen et al. PHN model:

- The membrane stimulation current equation was edited so that stimulation is phase-shifted by 20 ms and doubles in frequency after 5 seconds
- Ryanodine receptor and L-type calcium channel currents were increased by introducing a scaling factor of 1.5
- A Hill coefficient in the equation for the sodium-potassium pump current was increased from 1.5 to 4
- Initial conditions were changed for several of the variables solved using ordinary differential equations.

**Fig 3:**
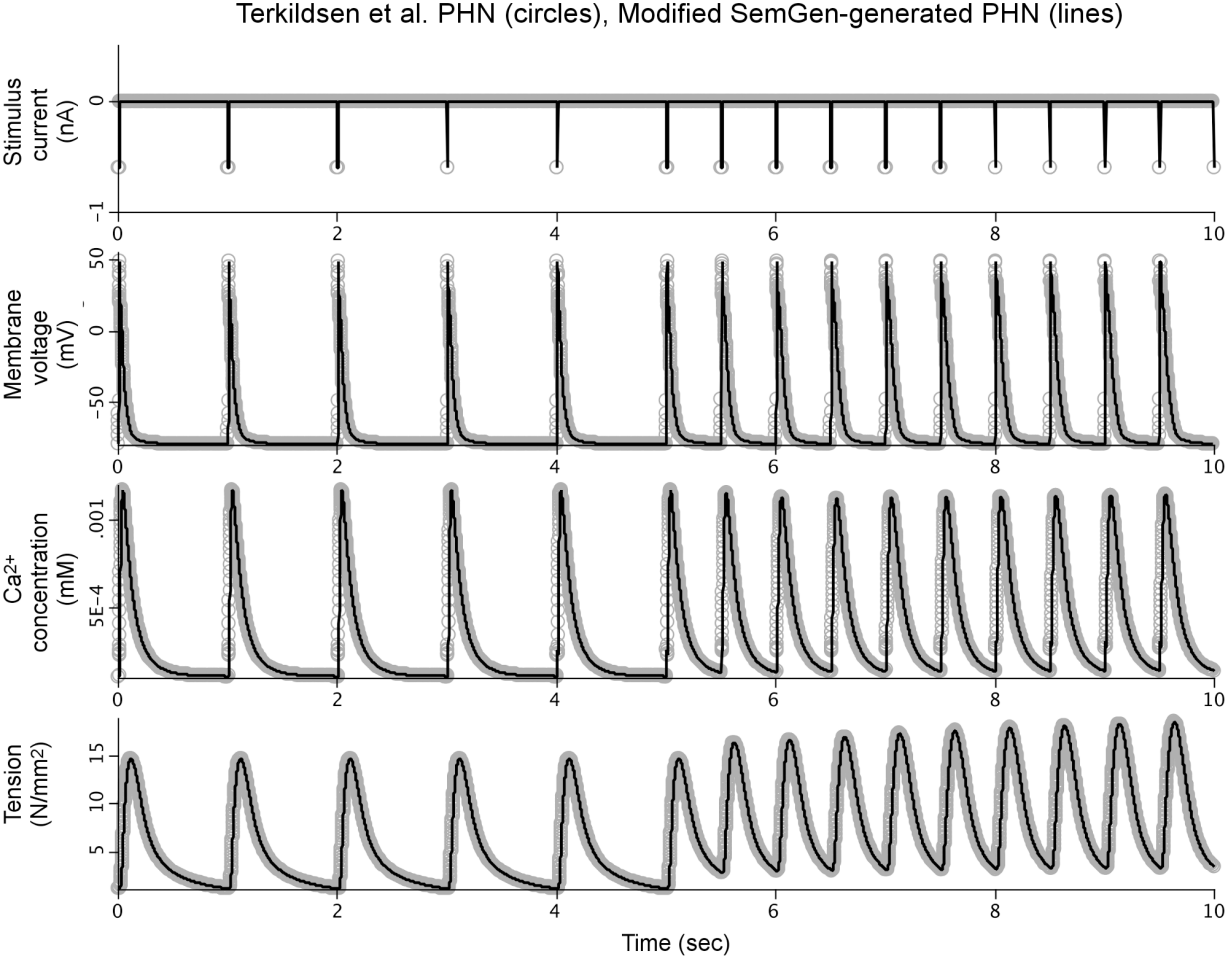
Comparison between simulation results from the original PHN model and the manually-modified SemGen-generated version. This modified SemGen-generated model includes the adjustments to equations and initial conditions that were introduced into the PHN model published by Terkildsen et al.

We manually incorporated these adjustments from the Terkildsen et al. PHN model into the simulation code of our SemGen-generated PHN model. Fig 3 compares the simulation results between this new version and the Terkildsen et al. version.

Because we did not resolve all the known biophysical overlap between the source models used in the PHN merge, the SemGen-generated version includes redundant representations of several biological components. For example, both representations of the sarcoplasmic reticulum (SR) and the diadic space (DS), including the calcium concentrations therein, are contained in the merged model. However, only the SR and DS from the Hinch model have any impact on tension generation dynamics. The Pandit representations of the SR and DS exist in a terminal branch of the model’s computational network and are effectively isolated from the model because the only fluxes that influence cytosolic calcium come from the Hinch SR and DS dynamics. The fluxes in the Pandit representation of the SR and DS, while they are computed based on cytosolic calcium concentration, do not in turn impact cytosolic calcium values. Due to biological redundancies such as these, the SemGen-generated model contains more computational code than the original PHN model: the SemGen-generated version contains 528 variables while the original contains 432.

### Pandit-Hinch-Tran Merge

To create the Pandit-Hinch-Tran (PHT) model, we merged our previously generated Pandit-Hinch model with the Tran model of myocyte tension development. Table 4 shows which semantic equivalencies SemGen automatically identified when generating the model, which unidentified equivalencies we had to manually specify, and which we left unresolved.

**Fig 4:**
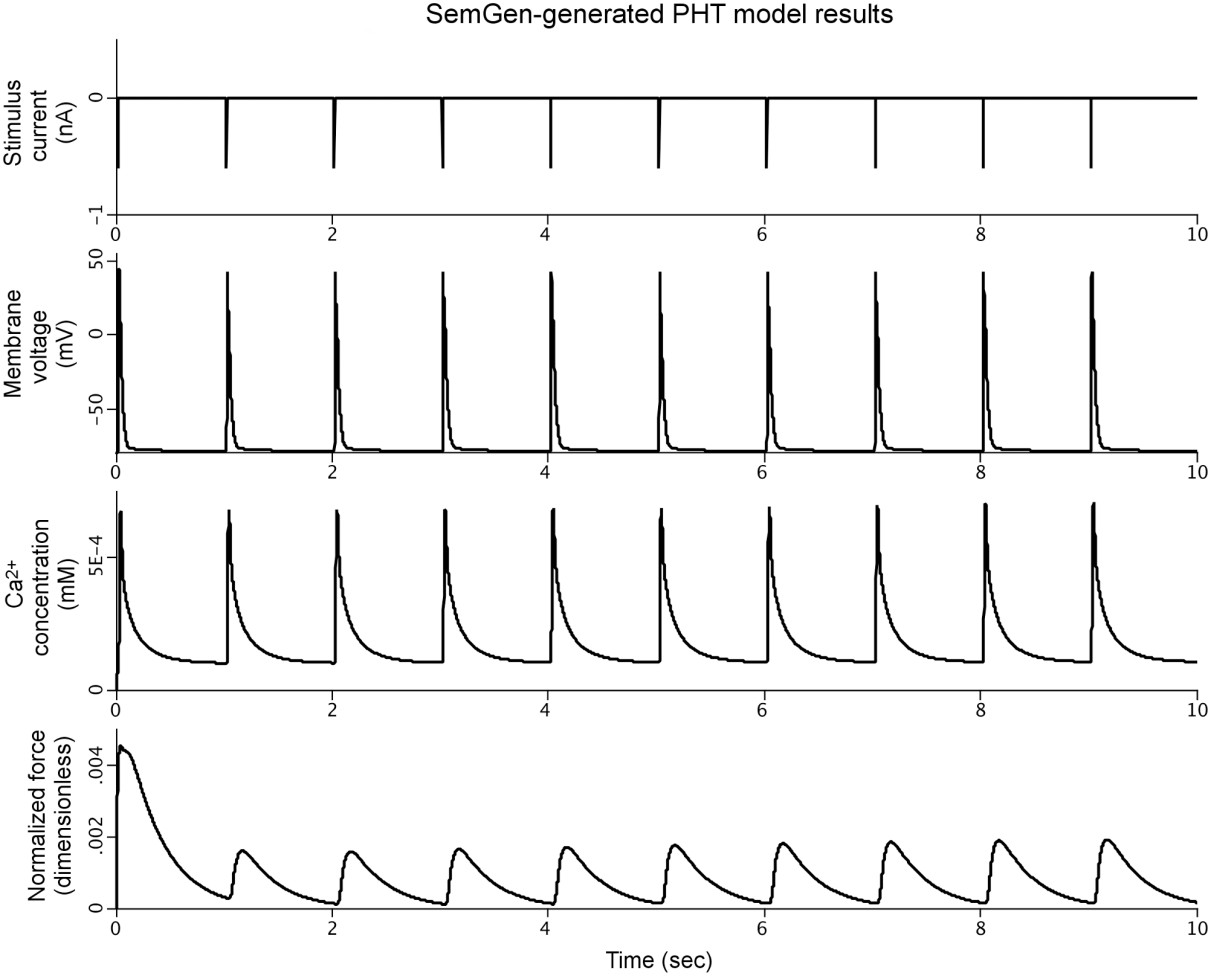
Simulation results for the PHT model demonstrating coupling of the stimulation current, membrane voltage, calcium transients, and cardiomyocyte force generation.

Fig 4 shows the simulation results for the SemGen-generated PHT model. All equation formulations and initial conditions from the original source models are preserved in this merged model; no manual, post-merge changes were made.

## Discussion

Our merging exercises demonstrate that we can use SemGen to successfully couple the Pandit, Hinch, Niederer and Tran models into integrated cardiomyocyte systems that appropriately link ion channel dynamics, calcium-induced calcium release, cross-bridge dynamics and tension generation. We were able to apply SemGen’s SAIM-style approach to model integration, and in the majority of cases, specify the connections between source models at the level of their biophysics, rather than their mathematics. We were thus able to avoid much of the manual encoding required for generating the original PHN model, including the burden of “mapping” CellML variables to each other. Our impromptu creation of the novel, integrated PHT model further demonstrates the flexibility and compositional power of the SAIM approach.

As described above, reproducing the numerical results of the Terkildsen et al. PHN model using our SemGen-generated version required the manual addition of several post-merge adjustments originally made by Terkildsen et al. Without these adjustments, the tension generated by the PHN model is relatively low (compare the bottom curves of Figs 2 and 3). The changes increase the peak of the calcium transient to elicit a higher tension response that is more comparable to the Niederer source model. Similarly, the normalized force generated by the PHT model (Fig 4, bottom curve) is relatively low compared to the force generated in the Tran source model. This is also because the cytosolic calcium levels generated by the Pandit-Hinch model are lower than those used in the Tran source model. Therefore, additional post-merge parameter changes would be required to align the assumptions about calcium levels in the components of the PHT model. These kinds of post-merge adjustments are an important part of a modeler’s workflow when merging models. Often, they require a clear understanding of the mathematical relations among variables, and a broad contextual knowledge about the assumptions and underlying biology behind the models. For example, source models may be parameterized under different experimental conditions and for different species. The post-merge edits, such as those we described above, represent subtle (and sometimes subjective) decision-making by human intelligence. It is not within the scope of SemGen to help automate this aspect of model merging. Rather, SemGen aims to make all of the straightforward connections, and do the “heavy lifting” of semantic resolution, merging and automatic code generation. Using our tool, the modeler can more easily move to the challenging and subtler decisions about assumptions, context, and adjustments to parameters that might be needed to produce a biologically sensible result.

### Time-Saving Benefits

While we have not yet performed formal tests to quantify the amount of time SemGen saves users when performing model merging tasks, the present study provides preliminary evidence, albeit anecdotal, illustrating its time-saving features. Based on estimates from the authors, the original Terkildsen et al. model required months to generate whereas our version required only weeks. Furthermore, the time required for us to assemble our version included the time required to annotate the models, and also to enhance and debug SemGen so that it correctly performed the model-model resolution, merging, and code-generation procedures. The actual time required to load in the annotated models, resolve their semantic overlap and output simulation code requires on the order of several minutes only. Indeed, authors KT, BEC and JHG were able to quickly generate an impromptu version of the PHT model during a 15-minute conference break—they already had the annotated Pandit and Hinch models available, and connecting them to the Tran model was simply a matter of annotating cytosolic calcium in the Tran model and then merging the Pandit-Hinch and Tran models in SemGen. We acknowledge that this process was likely facilitated by KT’s expert knowledge of the Pandit and Hinch models, nonetheless, we believe this impromptu composition is an important illustration of the time-saving benefits that are available through semantics-based model composition.

We also note that after creating our version of the PHN model, we were able to quickly generate a model variant that used the *epi*cardial rather than *endo*cardial version of the Pandit model. The epicardial version is very similar to the endocardial version, and includes many of the same variables and equations. We were therefore able to automatically import many of the annotations from the endocardial version and apply them to the epicardial version, merge the latter with the Hinch model, then merge the result with the Niederer model (using the same resolution decisions presented in Tables 2 and 3). The total amount of time required to generate this variant of the PHN model was approximately five minutes.

### Current Limitations and Solution Strategies

Our merging exercises illuminated several limitations in our approach that, if addressed, would further automate model composition in SemGen. As discussed in the Methods section and indicated in Tables 2–4, there were several points of semantic overlap between the models that SemGen did not recognize, requiring manual intervention during the merging process. From these examples, we have identified three primary impediments that account for the manual interventions we performed. These are presented below along with proposed solution strategies.

#### 1) Models represent semantically similar, but not identical, physical properties

As discussed in the Methods section, SemGen currently only recognizes semantic equivalency, not similarity. Therefore, to couple the Pandit and Hinch models in a biophysically consistent fashion, we manually introduced glue code into the Hinch model that represented ion channel activity in terms of electrical current rather than chemical concentration flow. This demonstrates how recognizing semantic similarity between models could accelerate the merging process. The activity of an ion channel expressed as electrical current is physically distinct from activity expressed as a molar flow rate, but the two properties are ontologically related: The terms ‘Charge flow rate’ and ‘Chemical concentration flow rate’, which represent the two properties used to express ion channel activity in this example, are both subclasses of *OPB*:*Flow rate property*. A semantic similarity scoring system that recognizes the close relationship between these terms could therefore be used to suggest inter-model links between ion channel activities, despite differences in implementations. We plan to investigate previously developed semantic similarity metrics [27,28] and eventually develop a scoring system within SemGen that will quantify the biological similarity of terms annotated with composite as well as singular annotations. We also plan to extend SemGen so that modelers can introduce glue code as needed during the merging process, rather than rely on the type of preemptive semantic alignment that we performed here.

Recognizing semantic similarity is also crucial for addressing differences in representational detail among models to be merged. In our merging exercise there were some cases where the same biophysical phenomena were simulated at different levels of granularity. For example, while the Hinch model represents troponin buffering using one chemical species for troponin, the Pandit model represents high- and low-affinity versions of troponin. Also, the Hinch model represents the SR as a single container while the Pandit model breaks up the SR into “junctional SR” and “network SR” sub-components. Enhancing SemGen to recognize such similarities and provide the means to resolve them would eliminate much of the redundant biophysical phenomena present in our merged models. One such enhancement would be to allow users to link custom physical entity terms such as “high-affinity troponin” to related classes from reference ontologies. This way, SemGen could recognize the close relationship between the two representations of troponin in the Pandit and Hinch models. Another enhancement would be to leverage the mereotopological information contained in the ontologies we use for annotation so that SemGen can recognize critical parthood relationships between biological structures represented in models.

Our merging exercises also showed us the importance of being able to recognize when two models simulate the same physical process, but with opposite directionality. For example, the Pandit model represents calcium-troponin buffering using a variable that simulates the association rate of calcium ion and troponin. The Hinch and Niederer models, on the other hand, represent the same process using a variable that simulates the *dissociation* rate of the calcium-troponin complex. SemGen currently recognizes equivalent physical processes by examining the physical property represented (electrical current, e.g.) and the physical entities that participate in the process as thermodynamic sources, sinks and mediators. Thus, if two models represent the same physical process but directionality is different such that the source entities for one model are the sinks for the other, SemGen does not yet recognize the equivalency. We plan to enhance SemGen so that it recognizes the close, inverse relationship between processes that have opposite directionality but are otherwise identical.

#### 2) Unavailable reference terms

During model annotation there were several physical entities for which we could not find suitable reference terms. We therefore used custom terms; however, SemGen cannot yet determine the biological relationship between composite annotations that include custom terms. There are several solutions to address this challenge. First, in some cases it may be prudent to send a term request to the appropriate ontology developers so that the reference term needed for annotation is added to the corpus of available reference ontologies. For example, we used a custom term for “diadic space” in our source models, and the developers of the FMA have said they would be willing to add this term to their ontology. Second, SemGen could potentially recognize the relationship between custom terms if they were linked to existing reference terms using structural and/or hierarchical relationships. For example, calcium-bound troponin (another custom term used in annotating our source models) could be linked to *CHEBI*:*calcium(2+)* and *PR*:*Troponin C*, *slow skeletal and cardiac muscles* using the *has_part* relation. Many examples of such linkages are found in SBML [6] models available in the BioModels database [29] because, as we also report here, reference terms that provide a precise biophysical definition for an annotated object are not always available. Previous studies within the SBML domain describe methods for quantifying the semantic similarity of model elements that are annotated using non-identity relations such as *has_part* [27]. In the future we plan to apply such methods to allow SemGen to recognize the relationship between custom terms. A third solution would be to establish a new knowledge resource that provides unique identifiers for whatever custom terms are created in curated SemSim models. In addition to providing a set of terms for molecular complexes (such as calcium-bound troponin), this knowledge resource would also provide a set of terms representing *functional*, as distinct from structural, anatomy since many anatomical entities used in biosimulation models are defined by their physiological function rather than their structural characteristics (such as the diadic space). Such terms could then be re-used during the annotation of new models.

#### 3) Recognizing equivalent constitutive properties

As mentioned in the Methods section, when annotating our source models we focused on model data structures that represent the properties of physical entities and processes. We only annotated constitutive properties, the third type of property, if a singular reference term was available in the OPB. Therefore, we had to manually specify the semantic equivalency between the rapid buffer coefficient used to represent calmodulin-calcium binding in the Pandit and Hinch models. To address this issue, we plan to enhance SemGen such that it can automatically recognize and/or propose annotations for constitutive properties as the annotation process proceeds and ontological information accumulates in the SemSim model. This will minimize the manual annotation of constitutive properties and still allow SemGen to recognize inter-model equivalencies among them. Accomplishing this will require substantial development of the OPB. Specifically, we must assert logical statements among the *OPB*:*Physical dependency* subclasses that indicate which physical properties are involved in which dependencies. Once this knowledge is asserted in the OPB, SemGen can use it to automatically identify appropriate annotations for constitutive properties based on how the property is used within the model’s computational dependency network.

Despite the challenges listed above, we were nonetheless able to semi-automatically merge our source models into integrated cardiomyocyte models that generated desired simulation results in terms of membrane dynamics, calcium dynamics and tension development. This illustrates that recognizing and resolving all points of semantic connection between merged models may not be required for creating an integrated system that meets a modeler’s needs. Even though we left several points of semantic connection unresolved and thus allowed redundant biological content in the merged system, this content did not impact the simulation results for our variables of interest; it is computationally isolated from the rest of the model and can be pruned without consequence. For example, although the merged PHN model includes all three representations of calcium-troponin buffering present in the source models, only the Hinch representation has any influence on the model because only it impacts calcium concentration. This arrangement was established when we resolved the equivalency for calcium concentration between the Pandit and Hinch models; by choosing the Hinch representation of this concentration, we effectively chose to use the Hinch representation of calcium-troponin buffering at that point because the equation that solves for calcium concentration uses the Hinch representation of the buffering rate. This example demonstrates the value of providing users with a clear description of how their semantic resolution choices will impact the formulation of the merged model. We anticipate that graph visualizations would be a useful addition for communicating the consequences of such resolution choices within SemGen. As resolution choices are made, visualizing the changes to the merged model’s computational dependency network and/or PhysioMap [30] could help users quickly ascertain exactly which parts of the source models will be preserved in the merged system and how they will be linked together computationally.

To summarize, we have applied semantics-based model composition to generate several versions of integrated cardiomyocyte models. Based on real-world modeling use cases, these exercises brought into relief the time-saving benefits of our approach and several important limitations. Addressing these limitations in the future will improve the accuracy of semantics-based model-to-model comparisons and further accelerate the model composition process. It is our intent to continue refinement of our approach and produce a robust set of modeling tools that significantly accelerates modeling work across research areas that employ biosimulation.

## References

[1] Neal ML, Cooling MT, Smith LP, Thompson CT, Sauro HM, Carlson BE, et al A reappraisal of how to build modular, reusable models of biological systems. PLoS Comput Biol. 2014/10/03 ed. 2014;10: e1003849 10.1371/journal.pcbi.1003849 25275523 PMC4183381

[2] Gennari JH, Neal ML, Galdzicki M, Cook DL. Multiple ontologies in action: Composite annotations for biosimulation models. J Biomed Inform. 2011;44: 146–154. 10.1016/j.jbi.2010.06.007 20601121 PMC2989341

[3] Beard DA, Neal ML, Tabesh-Saleki N, Thompson CT, Bassingthwaighte JB, Shimoyama M, et al Multi-scale modeling and data integration in the Virtual Physiological Rat Project. Ann Biomed Eng. 2012;40: 2365–2378. 10.1007/s10439-012-0611-7 22805979 PMC3463790

[4] Bechhofer S, van Harmelen F, Hendler J, Horrocks I, McGuinness DL, Patel-Schneider PF, et al. OWL Web Ontology Language Reference. In: W3C recommendation [Internet]. 2004. Available: http://www.w3.org/TR/owl-ref/.

[5] Cuellar AA, Lloyd CM, Nielsen PF, Bullivant DP, Nickerson DP, Hunter PJ. An overview of CellML 1.1, a biological model description language. Simulation. 2003;79: 740–747.

[6] Hucka M, Finney A, Sauro HM, Bolouri H, Doyle JC, Kitano H, et al The systems biology markup language (SBML): a medium for representation and exchange of biochemical network models. Bioinformatics. 2003;19: 524–531. 12611808 10.1093/bioinformatics/btg015

[7] Butterworth E, Jardine BE, Raymond GM, Neal ML, Bassingthwaighte JB. JSim, an open-source modeling system for data analysis. F1000Research. 2013;2: 288 10.12688/f1000research.2-288.v1 24555116 PMC3901508

[8] Neal ML, Gennari JH, Arts T, Cook DL. Advances in semantic representation for multiscale biosimulation: A case study in merging models. Pac Symp Biocomput. 2009: 304–315. 19209710 PMC2831637

[9] Terkildsen JR, Niederer S, Crampin EJ, Hunter P, Smith NP. Using Physiome standards to couple cellular functions for rat cardiac excitation-contraction. Exp Physiol. 2008;93: 919–929. 10.1113/expphysiol.2007.041871 18344258

[10] Niederer SA, Smith NP. A mathematical model of the slow force response to stretch in rat ventricular myocytes. Biophys J. 2007;92: 4030–4044. 17369410 10.1529/biophysj.106.095463 PMC1868992

[11] Pandit S V, Clark RB, Giles WR, Demir SS. A mathematical model of action potential heterogeneity in adult rat left ventricular myocytes. Biophys J. 2001;81: 3029–3051. 11720973 10.1016/S0006-3495(01)75943-7 PMC1301767

[12] Hinch R, Greenstein JL, Tanskanen AJ, Xu L, Winslow RL. A simplified local control model of calcium-induced calcium release in cardiac ventricular myocytes. Biophys J. 2004;87: 3723–3736. 15465866 10.1529/biophysj.104.049973 PMC1304886

[13] Niederer SA, Hunter PJ, Smith NP. A quantitative analysis of cardiac myocyte relaxation: a simulation study. Biophys J. 2006;90: 1697–1722. 16339881 10.1529/biophysj.105.069534 PMC1367320

[14] Tran K, Smith NP, Loiselle DS, Crampin EJ. A metabolite-sensitive, thermodynamically constrained model of cardiac cross-bridge cycling: Implications for force development during ischemia. Biophys J. 2010;98: 267–276. 10.1016/j.bpj.2009.10.011 20338848 PMC2808479

[15] Cook DL, Bookstein FL, Gennari JH. Physical Properties of Biological Entities: An Introduction to the Ontology of Physics for Biology. PLoS One. 2011;6: e28708 10.1371/journal.pone.0028708 22216106 PMC3246444

[16] Cook DL, Neal ML, Bookstein FL, Gennari JH. Ontology of physics for biology: representing physical dependencies as a basis for biological processes. J Biomed Semantics. 2013;4: 41 10.1186/2041-1480-4-41 24295137 PMC3904761

[17] Neal ML, Cook DL, Gennari JH. An OWL knowledge base for classifying and querying collections of physiological models: A prototype human physiome. International Conference on Biomedical Ontology. 2013: 16–21.

[18] Rosse C, Mejino JL V. A Reference Ontology for Bioinformatics: The Foundational Model of Anatomy. J Biomed Inform. 2003;36: 478–500. 14759820 10.1016/j.jbi.2003.11.007

[19] Hayamizu TF, Mangan M, Corradi JP, Kadin JA, Ringwald M. The Adult Mouse Anatomical Dictionary: a tool for annotating and integrating data. Genome Biol. 2005;6: R29 15774030 10.1186/gb-2005-6-3-r29 PMC1088948

[20] Bard J, Rhee SY, Ashburner M. An ontology for cell types. Genome Biol. 2005;6: R21.1–R21.5. 15693950 10.1186/gb-2005-6-2-r21 PMC551541

[21] Harris MA, Clark J, Ireland A, Lomax J, Ashburner M, Foulger R, et al The Gene Ontology (GO) database and informatics resource. Nucleic Acids Res. 2004;32: D258–61. 14681407 10.1093/nar/gkh036 PMC308770

[22] Natale DA, Arighi CN, Blake JA, Bult CJ, Christie KR, Cowart J, et al Protein Ontology: A controlled structured network of protein entities. Nucleic Acids Res. 2014;42: D415–D421. 10.1093/nar/gkt1173 24270789 PMC3964965

[23] Hastings J, De Matos P, Dekker A, Ennis M, Harsha B, Kale N, et al The ChEBI reference database and ontology for biologically relevant chemistry: Enhancements for 2013. Nucleic Acids Res. 2013;41: D456–D463. 10.1093/nar/gks1146 23180789 PMC3531142

[24] Brinkman RR, Courtot M, Derom D, Fostel JM, He Y, Lord P, et al Modeling biomedical experimental processes with OBI. J Biomed Semantics. 2010;1 Suppl 1: S7 10.1186/2041-1480-1-S1-S7 20626927 PMC2903726

[25] Garny A, Hunter PJ. OpenCOR: a modular and interoperable approach to computational biology. Front Physiol. Frontiers Media S.A.; 2015;6: 26 10.3389/fphys.2015.00026 25705192 PMC4319394

[26] Rice JJ, Wang F, Bers DM, de Tombe PP. Approximate model of cooperative activation and crossbridge cycling in cardiac muscle using ordinary differential equations. Biophys J. 2008;95: 2368–2390. 10.1529/biophysj.107.119487 18234826 PMC2517033

[27] Schulz M, Klipp E, Liebermeister W. Propagating semantic information in biochemical network models. BMC Bioinformatics. 2012;13: 18 10.1186/1471-2105-13-18 22289386 PMC3340317

[28] Schulz M, Krause F, Le Novère N, Klipp E, Liebermeister W. Retrieval, alignment, and clustering of computational models based on semantic annotations. Mol Syst Biol. 2011;7: 512 10.1038/msb.2011.41 21772260 PMC3159965

[29] Li C, Donizelli M, Rodriguez N, Dharuri H, Endler L, Chelliah V, et al BioModels Database: An enhanced, curated and annotated resource for published quantitative kinetic models. BMC Syst Biol. 2010;4: 92 10.1186/1752-0509-4-92 20587024 PMC2909940

[30] Cook DL, Neal ML, Hoehndorf R, Gkoutos G V, Gennari JH. Representing physiological processes and their participants with PhysioMaps. J Biomed Semantics. 2013;4 Suppl 1: S2 10.1186/2041-1480-4-S1-S2 23735231 PMC3632997

